# Signal enhancement in the output stage of the basal ganglia by synaptic short-term plasticity in the direct, indirect, and hyperdirect pathways

**DOI:** 10.3389/fncom.2013.00076

**Published:** 2013-06-19

**Authors:** Mikael Lindahl, Iman Kamali Sarvestani, Örjan Ekeberg, Jeanette Hellgren Kotaleski

**Affiliations:** ^1^Department of Computational Biology, School of Computer Science and Communication, KTH Royal Institute of TechnologyStockholm, Sweden; ^2^Department of Neuroscience, Karolinska InstituteStockholm, Sweden

**Keywords:** substantia nigra pars reticulata, short-term plasticity, basal ganglia, network model, subthalamic nucleus, globus pallidus, facilitation, depression

## Abstract

Many of the synapses in the basal ganglia display short-term plasticity. Still, computational models have not yet been used to investigate how this affects signaling. Here we use a model of the basal ganglia network, constrained by available data, to quantitatively investigate how synaptic short-term plasticity affects the substantia nigra reticulata (SNr), the basal ganglia output nucleus. We find that SNr becomes particularly responsive to the characteristic burst-like activity seen in both direct and indirect pathway striatal medium spiny neurons (MSN). As expected by the standard model, direct pathway MSNs are responsible for decreasing the activity in SNr. In particular, our simulations indicate that bursting in only a few percent of the direct pathway MSNs is sufficient for completely inhibiting SNr neuron activity. The standard model also suggests that SNr activity in the indirect pathway is controlled by MSNs disinhibiting the subthalamic nucleus (STN) via the globus pallidus externa (GPe). Our model rather indicates that SNr activity is controlled by the direct GPe-SNr projections. This is partly because GPe strongly inhibits SNr but also due to depressing STN-SNr synapses. Furthermore, depressing GPe-SNr synapses allow the system to become sensitive to irregularly firing GPe subpopulations, as seen in dopamine depleted conditions, even when the GPe mean firing rate does not change. Similar to the direct pathway, simulations indicate that only a few percent of bursting indirect pathway MSNs can significantly increase the activity in SNr. Finally, the model predicts depressing STN-SNr synapses, since such an assumption explains experiments showing that a brief transient activation of the hyperdirect pathway generates a tri-phasic response in SNr, while a sustained STN activation has minor effects. This can be explained if STN-SNr synapses are depressing such that their effects are counteracted by the (known) depressing GPe-SNr inputs.

## Introduction

An important question in neuroscience is to understand how synaptic signaling contributes to network function in the brain. The synapse, as a basic communication channel between neurons, has classically been viewed as providing information of whether a pre-synaptic neuron has spiked or not. However, the effect of the synaptic signal varies with previous activity pattern either at one or at both sides of the synapse, and these modifications include short-term- to long-term plasticities, which together span from milliseconds up to months (Abbott and Regehr, 2004). The activity history of the synapse thus becomes important in determining its current function in neural circuits. The ability of synapses to perform non-linear transformations of signals over time makes them crucial components enabling a diverse set of circuit functions in the nervous system such as gain control, information filtering, coincident detection, short term- and long term memory (Abbott and Regehr, 2004; Deng and Klyachko, 2011).

Synapses with short-term plasticity are frequent in the basal ganglia, a group of subcortical nuclei involved in action selection and procedural learning (Mink, 1996; Redgrave et al., 1999; Grillner et al., 2005), but still the functional role of these synapses remains poorly understood. Synapses that undergo frequency dependent facilitation and depression on the time scale of hundred milliseconds can be found in several parts of the basal ganglia (Hanson and Jaeger, 2002; Sims et al., 2008; Connelly et al., 2010; Gittis et al., 2010; Planert et al., 2010). Many computational models of the basal ganglia exist. However, with regard to how synaptic connectivity is represented they can roughly be divided into two categories, those without synaptic plasticity and those with long term synaptic plasticity (see, e.g., Bar-Gad et al., 2000; Terman et al., 2002; Humphries et al., 2006; Leblois et al., 2006; O'Reilly, 2006; Houk et al., 2007; Kumar et al., 2011). Although synaptic short-term plasticity is prominent in the basal ganglia, it has not been included in computational models of the basal ganglia.

The basal ganglia nuclei have been suggested to be involved in action selection, working memory representation, sequence learning, and reinforcement learning of appropriate actions (Chakravarthy et al., 2010; Kamali Sarvestani et al., 2011). The excitatory input to striatum, the basal ganglia main input stage, arrives from nearly all parts of cerebral cortex (Gerfen and Bolam, 2010) as well as midline, intralaminar, mediodorsal and ventral lateral, and anterior thalamus (Groenewegen, 1988; Smith et al., 2004). The basal ganglia output targets are also midline, intralaminar and mediodorsal thalamus as well as ventral lateral thalamus, involved in cortical planning and execution of motor behavior (Smith et al., 2004). Another major output are areas in the brainstem such as the superior colliculus, which generates eye and head movements, and pedunculopontine nucleus, involved in orienting of body movements (Gerfen and Bolam, 2010) and muscle tone control (Takakusaki et al., 2004). A third important output from substantia nigra reticulata (SNr) is to neighboring neurons in the substantia nigra compacta (SNc) were SNr efficiently controls the activity of SNc dopaminergic neurons (Tepper and Lee, 2007). Three major pathways, converging on the basal ganglia output stages have been described, the direct, indirect and hyperdirect pathways (Nambu, 2008). Specifically the output nuclei receive inputs from striatal medium spiny neurons expressing dopamine receptor D1 (MSN D1) in striatum (the direct pathway) and from MSNs expressing dopamine receptor D2 (MSN D2) in striatum via globus pallidus externa (GPe) and the subthalamic nucleus (STN) (the indirect pathway), and directly from cortex via the STN (the hyperdirect pathway). The temporal and spatial integration of these three pathways onto the output nuclei determine the ultimate effect basal ganglia signaling has on the behavioral response.

The relative contribution of signals from striatum, GPe and STN to activity changes in basal ganglia output nuclei, such as SNr, is not understood in detail, nor how changes in SNr activity facilitates or inhibits spiking behavior in target areas. SNr has an inhibitory control of thalamic and brainstem areas (Deniau et al., 2007) and a standard view is that decreased SNr activity promote actions whereas increase activity suppress actions (Mink, 1996; Redgrave et al., 1999). Recent experimental data support this view and show how SNr neurons increase and decrease their activity in relation to actions (Fan et al., 2012). SNr activity can potentially be decreased by either increased inputs from MSN D1 or GPe, whereas the SNr activity can be increased either through disinhibition via GPe or by increased excitatory input from STN. It still remains an open question which inputs are responsible for the observed increases and decreases in activity in SNr seen in experiments (Fan et al., 2012). Most of these inputs to SNr are in addition displaying short term plasticity and are thus modulated with activity over time.

Here we build a quantitative computational model of the striatal, pallidal, and subthalamic inputs to the basal ganglia output stage, SNr, assuming biologically plausible neuron dynamics, synaptic conductances and projection patterns, as well as appropriate firing patterns in the pre-synaptic neurons. We quantify the relative contribution of the direct, indirect and hyperdirect pathways for increasing and decreasing the activity in SNr as well as for the temporal integration of the inputs. We hypothesize that facilitating striato-nigral and depressing pallido-nigral- and subthalamo-nigral synapses in a significant way determine the relationship between timing and strength of input signals in SNr. We find that the direct pathway is responsible for decreased activity in SNr whereas pauses in GPe are preferentially responsible for the increased activity in SNr neurons. By assuming that STN synapses are depressing we can explain experiments showing that STN input, on a slower time scale, act as less potent source for changing activity in SNr compared to brief transient (ms) STN activity. Simulations are used to investigate how the rate coding may change with duration of the input signal and the proportion of active neurons. We also show how facilitating and depressing synapses buffer against fluctuations in input background activity.

## Materials and methods

### Neuronal firing rates

The characteristic of MSN activity *in vivo* (in both anesthetized and un-anesthetized preparations) is a low frequency firing interrupted by bursts (Wilson, 1993). The basal firing rate for MSNs ranged in simulations between 0.01 and 2.0 Hz while spike frequency during the bursts ranged between 17 and 48 Hz (Miller et al., 2008). The length of the burst was set to 500 ms which is in line with experiments showing that MSNs usually burst for 100–1000 ms (Miller et al., 2008; Gage et al., 2010).

GPe neurons fire tonically at high frequency, interrupted by bursts and pauses (Jaeger and Kita, 2011; Kita and Kita, 2011) and have been reported to fire, *in vivo* in rodents, at 17 Hz (Gage et al., 2010), 26 Hz (Walters et al., 2007), 29 Hz (Kita and Kita, 2011), 32 Hz (Urbain et al., 2000), 36 Hz (Ruskin et al., 1999), and 52 Hz (Celada et al., 1999). Here the GPe basal firing rate is required to be around 30 Hz.

STN neurons were required to have a basal firing rate around 10 Hz which is in accordance with *in vivo* recordings in rat: 6 Hz (Walters et al., 2007), 10 Hz (Farries et al., 2010), 11 Hz (Fujimoto and Kita, 1993), and 13 Hz (Paz et al., 2005).

The basal firing rate of SNr neurons, with MSN input arriving at 0.1 Hz, GPe input arriving at around 30 Hz and an STN background of 10 Hz, was required to be around 30 Hz which is in the range of reported values from *in vivo* recordings in rat: 22 Hz (Zahr et al., 2004), 24 Hz (Walters et al., 2007), 24–27 Hz (Maurice et al., 2003), and 29 Hz (Gernert et al., 2004).

### Neuron modeling approach

To model the SNr, GPe, and STN neurons we have chosen the adaptive exponential integrate and fire model (Brette and Gerstner, 2005). It has few parameters, simplifying the estimation of them from limited amount of experimental data, as compared to more complicated biophysical models with up to hundred or more parameters. The model can capture the spike initiation and upstroke, as well as subthreshold resonance and adaptation of neural activity. It can be tuned to reproduce simulated subthreshold and spiking behaviors that are very similar to *in vitro* and *in vivo* neuronal voltage responses. The model equations are explained below, where *V* is the membrane potential and *w* is the contribution of the neurons slow currents:

(1) $$\begin{array}{l} C\frac{dV}{dt}=-g_{L}(V-E_{L})+g_{L}\Delta_{T}\exp\left(\frac{V-V_{T}}{\Delta_{T}}\right)-w+I\\ \;\tau_{w}\frac{dw}{dt}=a(V-E_{L})-w\\ \text{ if }V>V_{peak}\text{ then }V=V_{r}\text{ and }w=w+b \end{array}$$

Here *C* is the capacitance, *g*_*L*_ is the leak conductance, *E*_*L*_ and *V*_*T*_ are the resting and threshold potentials, Δ_*T*_ is the slope factor of the spike upstroke, *I* is a current source and represents injected current *I*_inj_ and/or synaptic contributions *I*_syn_, τ_*w*_ and *a* are respectively the time constant and the subthreshold adaptation of the recovery current *w*. When the membrane potential *V* reaches the cut off *V*_peak_ it is reset to *V*_*r*_ and then the recovery current *w* is increased with *b*.

### SNr neuron model

**Figure 1:**
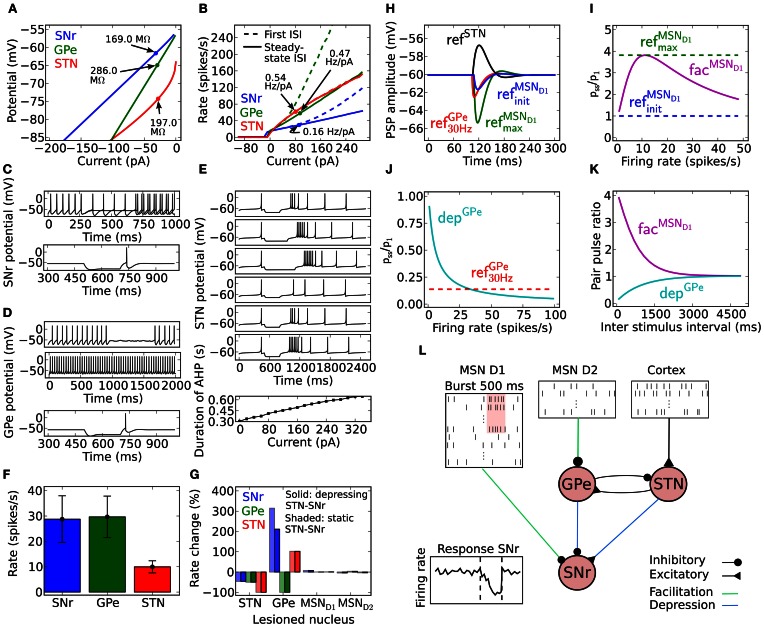
**Model properties. (A)** Steady-state current voltage relationship for SNr (blue), GPe (green) and STN (red). **(B)** Current frequency relation for SNr (blue), GPe (green), and STN (red). **(C)** SNr neuron properties. Upper panel: a difference of 5 pA in the injected hyperpolarizing current during the interval 250–750 ms can switch the SNr neuron from spiking above 1 Hz to silent. Lower panel; rebound spike is triggered upon release of a hyperpolarizing current provided for 200 ms. **(D)** GPe neuron properties. Membrane oscillations/spikes are revealed close to threshold by added noise (first panel). Current injection leads to regular high frequency spiking (second panel). Hyperpolarization induced spike (third panel). **(E)** STN neuron properties. First to third panel; increasing duration of −70 pA hyperpolariazing current (300, 450, and 600 ms) increases the length of the resulting burst. Fourth to sixth panel; increased strength of 300 ms hyperpolarizing currents (−40, −70, and −100 pA) lead to increased length of the hyperpolarization induced burst. Seventh panel; the amplitude of a 500 ms duration depolarizing current pulse has a linear relation to the afterhyperpolarization duration upon release of the injected current, defined from the end of the current pulse to first spike. **(F)** Basal firing rate for each population. The error bars show the standard deviation of individual firing rates of neurons in the population. **(G)** Firing rate change in SNr, GPe, and STN compared to basal rate **(F)** when removing GPe, STN, MSN D1, or MSN D2 nuclei. Solid bars show the result for depressing STN synapses in SNr and shaded bars the results using static STN synapses in SNr. **(H)** Post-synaptic potential (PSP) in SNr for GPe ref^GPe^_30 Hz_ (red), MSN D1 ref^MSN_*D*1_^_init_ (blue), MSN D1 ref^MSN_D1_^_max_ (green), and STN ref^STN^ (black) synapses. For further explanations see Materials and Methods. **(I)** Relation between synaptic steady-state IPSP (P_ss_) amplitude in SNr and initial response (P_1_) for different spike frequencies for ref^MSN_*D*1_^_init_ (blue), ref^MSN_D1_^_max_ (green), and fac^MSN_*D*1_^ (magenta) MSN D1 synapses in SNr. **(J)** Same as in **(I)** but for a ref^GPe^_30 Hz_ (red) and dep^GPe^ (cyan) GPe synapses in SNr. **(K)** Recovery from facilitation and depression respectively for the MSN D1 and GPe synapse in SNr. **(L)** Illustration of the complete network model, with emulated input from 15000 MSN D1 and 15000 MSN D2 as well as a summed backround input of 189 Hz from cortex to STN neurons. In the illustration a subpopulation of MSN D1 bursts and this leads to a delayed decrease of activity in SNr.

Without any synaptic input SNr neurons fire tonically at membrane potentials above −54 mV (Richards et al., 1997; Atherton and Bevan, 2005; Chuhma et al., 2011). The autonomous firing is caused by a sodium dependent TTX insensitive inward current activated above −60 mV and a TTX sensitive current activated close to spike threshold. It also has an outward SK channel mediated current responsible for the spike afterhyperpolarization and the precise regular autonomous spiking (Atherton and Bevan, 2005; Zhou et al., 2008). Below we list the quantitative properties of the SNr neuron that are captured with the model:

1. Current voltage relation in the range −80 to −65 mV to be compatible with an input resistance in the range of 80–400 MΩ (see Figure 1A; Nakanishi et al., 1997; Richards et al., 1997; Lee and Tepper, 2007a,b; Chuhma et al., 2011).
2. Current frequency relation to be 0.08–0.2 Hz/pA in the range of 0–300 pA (see Figure 1B; Nakanishi et al., 1987b; Richards et al., 1997).
3. From holding potential at just below spike threshold, small changes around 5 pA in injected current are sufficient to bring the neuron from silent to repetitive firing (see Figure 1C; Atherton and Bevan, 2005).
4. Silent below −54 mV (Richards et al., 1997; Atherton and Bevan, 2005; Chuhma et al., 2011).
5. Rebound spike upon release from hyperpolarization (see Figure 1C; Nakanishi et al., 1987b, 1997).

The resulting SNr neuron model parameters are listed in Table 1. To capture the rebound spike induced after injection of a hyperpolarizing current (Nakanishi et al., 1987b, 1997) the level of subthreshold adaptation *a* was set to 3 nS and the time constant τ_*w*_ to 20 ms. This also contributed to achieving a model with characteristic afterhypolarization (Atherton and Bevan, 2005) and a positive *a* ensured that the modeled SNr neuron went from silent to spiking at above 1 Hz by a small change in injected current (Atherton and Bevan, 2005). The SNr neuron's steady-state I–V relation was then produced by setting *g*_*L*_ to 3 nS (Nakanishi et al., 1987b; Richards et al., 1997; Atherton and Bevan, 2005; Zhou et al., 2008). Near spike initiation the adaptive exponential integrate and fire model can approximate the upstroke and thus the voltage speed/acceleration of the action potential (Platkiewicz and Brette, 2010). For the modeled SNr neuron to go from silent to spiking at approximately −54 mV (Richards et al., 1997; Atherton and Bevan, 2005; Chuhma et al., 2011) and having spike threshold at −52 mV (Richards et al., 1997), defined as when the rate of rise is 10.2 mV/ms, the resting and threshold potentials and slope factor, *E*_*L*_, *V*_*T*_, and Δ_*T*_ were respectively estimated to −55.8, −55.2, and 1.8 mV. Note, the action potential threshold was measured when the rate of rise was 5% of max in Richards et al. (1997) which we estimated to 10.2 mV/ms from a sigmoid fit of the upstroke of an action potential. The capacitance *C* was set to 80 pF (Nakanishi et al., 1997) and the summed recovery current contribution, *b*, at spike reset was set to 200 pA to get the frequency acceleration and the spike frequency adaptation (Nakanishi et al., 1987b; Richards et al., 1997) of the SNr neuron. With the spike voltage reset, *V*_*r*_, at −65 mV and spike cut off, *V*_peak_, at 20 mV we got an after hyperpolarization and spike amplitude in accordance with literature (Lee and Tepper, 2007b). *I*_inj_ = *I*_*in vitro*_ was set to 15 pA to shift the current- voltage and frequency curves along the current axis, such that the neuron fired without any synaptic input around 14 Hz (see Figures 1A,B) which is in range of measured mean values in experiments with rat/mice slice preparations 7 Hz (Richards et al., 1997), 9–13 Hz (Atherton and Bevan, 2005), 16 Hz (Nakanishi et al., 1997), 16 Hz (Chuhma et al., 2011), and 16–20 Hz (Lee and Tepper, 2007b). To obtain the current- frequency and voltage curves in Figures 1A,B *I*_*in vitro*_ was successively changed. In the network simulations *I*_inj_ = *I*_*in vivo*_ was set to 254 pA to obtain around 30 Hz base line firing rate with full synaptic connectivity in the network model (see Figure 1F).

**Table 1:** **SNr neuron model parameters**.

**Name**	**Value**	**Description**
*a*	3 nS	Subthreshold adaptation
*b*	200 pA	Spike-triggered adaptation
*C*	80 pF	Membrane capacitance
Δ_*T*_	1.8 ms	Slope factor of spike upstroke
*E*_*L*_	−55.8 mV	Leak reversal potential
*g*_*L*_	3 nS	Leak conductance
*I*_*in vitro*_	15 pA	*I*_inj_ to obtain *in vitro* firing rate without synaptic input
*I*_*in vivo*_	254 pA	*I*_inj_ to obtain *in vivo* firing rate with synaptic input
τ_*w*_	20 ms	Adaptation time constant
*V*_peak_	20 mV	Spike cut off
*V*_*r*_	−65mV	Spike reset
*V*_*T*_	−55.2 mV	Threshold potential

### GPe neuron model

Several different types of neurons in GPe have been reported. They have been classified into subgroups based on electrophysiological properties such as rebound firing, membrane resistance, current-frequency relation, hyperpolarizing induced sag, and firing patterns (Kita and Kitai, 1991; Nambu and Llinaś, 1994; Cooper and Stanford, 2000; Bugaysen et al., 2010). However, in an exhaustive modeling and experimental study, it was showed that the properties of the GPe neurons vary in a continuous space without any clear division into subtypes (Günay et al., 2008). Thus, it is not clear how to come up with one model of the GPe neuron. Our approach was to create a GPe neuron model which showed general dominant characteristics of GPe neurons stated below:

1. Current voltage relation in the range −80 mV to −65 mV to be compatible with an input resistance in the range of 90–560 MΩ (see Figure 1A; Cooper and Stanford, 2000; Bugaysen et al., 2010; Chuhma et al., 2011).
2. Current frequency relation to be 0.2–0.6 Hz/pA in the range of 0–300 pA (see Figure 1B; Cooper and Stanford, 2000; Bugaysen et al., 2010).
3. Membrane oscillations close to spike threshold causing irregular firing and regular firing at higher depolarizing currents (see Figure 1D; Nambu and Llinaś, 1994; Cooper and Stanford, 2000).
4. Silent below −53 mV (Bugaysen et al., 2010; Chuhma et al., 2011).
5. Rebound spike upon release from hyperpolarization (see Figure 1D; Nambu and Llinaś, 1994; Cooper and Stanford, 2000).

The resulting GPe neuron model parameters are listed in Table 2. The hyperpolarization triggered spike (Nambu and Llinaś, 1994; Cooper and Stanford, 2000) was captured by setting the subthreshold adaptation *a* to 2.5 nS and the time constant τ_*w*_ to 20 ms. Steady-state current voltage relation of the GPe neuron was then produced by letting *g*_*L*_ be 1.0 nS (Cooper and Stanford, 2000; Bugaysen et al., 2010). The capacitance *C* was set to 40 pF (Cooper and Stanford, 2000). Note that with these parameters a GPe neuron exhibits subthreshold oscillations close to rheobase current (the minimal current necessary to elicit a spike). Touboul and Brette (2008) showed that whether an adaptive exponential integrate and fire neuron model exhibit oscillations close to spike threshold depends on the parameters *a*, *C*, *g*_*L*_, and τ_*w*_ and occurs when equations 2 and 3, with τ_*m*_ = *C*/*g*_*L*_, are fulfilled. For the modeled GPe neuron to go from silent to spiking at approximately −53 mV (Bugaysen et al., 2010; Chuhma et al., 2011) and having a spike threshold at −43 mV (Bugaysen et al., 2010), defined as when the acceleration of the membrane potential reaches 50% of its max, estimated to 1270 mV/ms^2^ from Bugaysen et al. (2010), the resting and threshold potentials and the slope factor, *E*_*L*_, *V*_*T*_ and Δ_*T*_ were set to respectively −55.1, −54.7, and 1.7 mV. The summed recovery current contribution, *b*, at spike reset was set to 70 pA, to mimick the frequency acceleration and the spike frequency adaptation of the GPe neuron (Nambu and Llinaś, 1994; Cooper and Stanford, 2000; Bugaysen et al., 2010). With the spike voltage reset, *V*_*r*_, at −60 mV and spike cut off, *V*_peak_, at 15 mV we got an after hyperpolarization and spike amplitude in accordance with literature (Cooper and Stanford, 2000). *I*_inj_ = *I*_*in vitro*_ was set to 5 pA to move the current- voltage- and frequency-curves along the current axis, such that the neuron fired around 15 Hz without any synaptic input (see Figures 1A,B) which is in range of measured mean values in experiments with rate slice preparations 8–14 Hz (Cooper and Stanford, 2000) and 4–18 Hz (Bugaysen et al., 2010). To get the current- frequency and voltage curves in Figures 1A,B *I*_*in vitro*_ was successively changed. In the network simulations *I*_inj_ = *I*_*in vivo*_ was set to 47 pA to obtain around 30 Hz base line firing rate with full synaptic connectivity in the network model (see Figure 1F).

**Table 2:** **GPe neuron model parameters**.

**Name**	**Value**	**Description**
*a*	2.5 nS	Subthreshold adaptation
*b*	70 pA	Spike-triggered adaptation
*C*	40 pF	Membrane capacitance
Δ_*T*_	1.7 ms	Slope factor of spike upstroke
*E*_*L*_	−55.1 mV	Leak reversal potential
*g*_*L*_	1 nS	Leak conductance
*I*_*in vitro*_	5 pA	*I*_inj_ to obtain *in vitro* firing rate without synaptic input
*I*_*in vivo*_	47 pA	*I*_inj_ to obtain *in vivo* firing rate with synaptic input
τ_*w*_	20 ms	Adaptation time constant
*V*_peak_	15 mV	Spike cut off
*V*_*r*_	−60 mV	Spike reset
*V*_*T*_	−54.7 mV	Threshold potential

(2) $$0>\frac{\tau_{m}}{\tau_{w}}-\frac{a}{g_{L}}$$

(3) $$0>\frac{\tau_{m}}{4\tau_{w}}{\left(1-\frac{\tau_{w}}{\tau_{m}}\right)}^{2}-\frac{a}{g_{L}}$$

### STN neuron model

The parameters for the model of the STN neuron were chosen such that it got some of the characteristic properties of STN neurons (Bevan and Wilson, 1999; Bevan et al., 2000). *In vitro* and in the absence of synaptic input, STN neurons exhibit autonomous rhythmic single-spike activity that is generated by voltage-dependent Na (Nav) channels and can fire at 250 Hz following current injection (Bevan and Wilson, 1999). We requested the following quantitative properties of the STN neurons:

1. Current voltage relation in the range −80 to −70 mV to be compatible with an input resistance in the range of 150–250 MΩ (see Figure 1A; Nakanishi et al., 1987a; Beurrier et al., 1999; Loucif et al., 2008).
2. Current frequency relation to be 0.4–0.8 Hz/pA in the range of 0–300 pA (see Figure 1B; Bevan and Wilson, 1999; Hallworth et al., 2003).
3. Duration of afterhypolarization after a brief depolarization around 500 ms should depend upon injected current strength (see Figure 1E; Bevan and Wilson, 1999).
4. Silent below −64 mV (Kass and Mintz, 2006).
5. Depolarizing the neuron below −70 mV for a certain period should lead to a rebound burst (Figure 1E; Bevan et al., 2000; Hallworth et al., 2003).

The resulting STN neuron model parameters are listed in Table 3. To account for the hyperpolarization activated inwards current responsible for rebound bursts, the subthreshold adaptation *a* was set to 0.3 nS below −70 mV with τ_*w*_ to 333 ms, such that $333\dot{w}=0.3\;(V+70)-w$, and to get minimal spike frequency adaptation (Bevan and Wilson, 1999) *a* was was set to 0 nS above −70 mV, such that $333\dot{w}=-w$. The STN neuron's steady-state current-voltage relation was captured by setting *g*_*L*_ to 10.0 nS (Nakanishi et al., 1987a; Beurrier et al., 1999). To get resting membrane potential at −64 mV (Kass and Mintz, 2006) and a spike threshold at −35 mV, when the acceleration of membrane potential is 50 mV/ms^2^ (Farries et al., 2010), the resting and threshold potentials, and the slope factor, *E*_*L*_, *V*_*T*_, and Δ_*T*_ were respectively set to −80.2, −64.0, and 16.2 mV. To capture the characteristic delayed afterhypolarization caused by increased current injection (Bevan and Wilson, 1999) as well as the spike frequency acceleration (Bevan and Wilson, 1999; Hallworth et al., 2003) the capacitance, *C*, the summed recovery current contribution, *b*, at spike reset and the spike voltage reset, *V*_*r*_, was respectively set to 60 pF, 0.05 pA, and −70 mV. The hyperpolarization induced bursts (Figure 1E; Bevan et al., 2000; Hallworth et al., 2003) were captured by resetting *V* following a spike to *V*_*r*_ + max(*w* × −10, 10) if *w* < 0 and else to *V*_*r*_. A similar modification to the spike reset point has been done by Izhikevich (2003). With the spike cut off, *V*_peak_, at 15 mV we got a spike amplitude in accordance with literature (Beurrier et al., 1999). *I*_inj_ = *I*_*in vitro*_ was set to 6 pA to shift the current- voltage and frequency curves along the current axis, such that the neuron fired without any synaptic input around 10 Hz (see Figures 1A,B) which is in range of measured mean values in experiments with rate slice preparations, 6 Hz (Baufreton et al., 2005), 8 Hz (Wilson et al., 2004), 8 Hz (Loucif et al., 2008), 10 Hz (Farries et al., 2010) 12 Hz (Hallworth et al., 2003). To obtain the current- frequency and voltage curves in Figures 1A,B *I*_*in vitro*_ was successively changed. In the network simulations *I*_inj_ = *I*_*in vivo*_ was also set to 6 pA to obtain around 10 Hz base line firing rate with full synaptic connectivity in the network model (see Figure 1F).

**Table 3:** **STN neuron model parameters**.

**Name**	**Value**	**Description**
*a*	0.3 nS	Subthreshold adaptation (below −70) otherwise equal to 0
*b*	0.05 pA	Spike-triggered adaptation
*C*	60 pF	Membrane capacitance
Δ_*T*_	16.2 ms	Slope factor of spike upstroke
*E*_*L*_	−80.2 mV	Leak reversal potential
*g*_*L*_	10 nS	Leak conductance
*I*_*in vitro*_	6 pA	*I*_inj_ to obtain *in vitro* firing rate without synaptic input
*I*_*in vivo*_	6 pA	*I*_inj_ to obtain *in vivo* firing rate with synaptic input
τ_*w*_	333 ms	Adaptation time constant
*V*_peak_	15 mV	Spike cut off
*V*_*r*_	−70 mV	Spike reset
*V*_*T*_	−64.0 mV	Threshold potential

### Network model

The model network consists of a population of SNr, GPe, and STN neurons receiving emulated inhibitory synaptic inputs from MSN D1, MSN D2 and cortex with a spike frequency as seen in experiments. The temporal distribution of the spikes was assumed to derive from an uncorrelated Poisson process. The synaptic inputs and neuron population sizes used are listed in Table 4, and are in accordance with experiments (Oorschot, 1996). To account for variability in mean firing rate of neurons, seen in experiments, the firing rate of neurons in SNr, GPe, and STN were Gaussian distributed with a standard deviation of 0.2 of respectively each nucleus mean *in vitro* firing rate. The distributions were created by varying the injected current for each of the neurons in a population.

**Table 4:** **Summary of network properties**.

**Name**	**Value**	**Description**
*N*_MSN__*D*1_	15,000	Number of MSN D1 inputs
*N*_MSN__*D*2_	15,000	Number of MSN D2 inputs
*N*_SNr_	300	Size of SNr population
*N*_GPe_	300	Size of GPe population
*N*_STN_	100	Size of STN population
*v*_MSN_*D*1__	0–47 Hz	Firing rate interval of individual MSN D1 neurons
*v*_MSN_*D*2__	0–47 Hz	Firing rate interval of individual MSN D2 neurons
*v*_STN_	189 Hz	Baser rate of external poisson type excitatory input to STN
*N*_MSN_*D*1_ − SNr_	500	Number of MSN D1 connecting to each SNr neuron
*N*_GPe − SNr_	32	Number of GPe connecting to each SNr neuron
*N*_STN − SNr_	30	Number of STN connecting to each SNr neuron
*N*_MSN_*D*2_ − GPe_	500	Number of MSN D2 connecting to each GPe neuron
*N*_STN − GPe_	30	Number of STN connecting to each GPe neuron
*N*_GPe − GPe_	30	Number of GPe reciprocal connections
*N*_GPe − STN_	30	Number of GPe connecting to each STN neuron

### Connectivity in the network

Synaptic parameters such as conductances and projection patterns are constrained by experimental data (Tables 4, 5). Below we first estimate the connectivity in the network starting with MSN D1 to SNr.

1. Striatal fibers entering SNr follow the dendritic course of single SNr neurons (Rinvik and Grofová, 1970; Schwyn and Fox, 1974; Tokuno et al., 1990) and the axons arborize in clusters along the way (Wu et al., 2000). Based on Miller (2007) (pp 21–28) we assume that a single axon from an MSN makes 20 synaptic contacts upon a single SNr neuron, similarly as for globus pallidus interna (GPi). We modeled this by assuming that the synaptic efficacy of an MSN-SNr connection in the model is the sum of the efficiency of all synaptic contacts that a pre-synaptic neuron makes onto a post-synaptic neuron.
2. The upper bound of the number of synapses an MSN gives off in SNr is 192 (Wu et al., 2000). By dividing 192 by 20, which was the number of synaptic contacts upon one SNr, we estimate that an MSN on average contacts around 10 SNr neurons.
3. Striatum in rat contains 2.8 million MSNs (Oorschot, 1996) and half of these, 1.4 million, belong to the direct pathway and project to SNr (Gerfen et al., 1990) with a subpopulation also sending collaterals to the much smaller endopeduncular nucleus (EP) (homologous to GPi in rat) (Wu et al., 2000).
4. SNr contains 26,000 neurons and EP contains 3200 (Oorschot, 1996) and the ratio between number of MSNs and SNr neurons becomes around 50 (divide 1.4 million by 26,000; assuming EP only receives SNr collaterals)
5. Combining the information in 2 and 4 suggests that each SNr can receive input from up to 500 MSNs.

**Table 5:** **Basic synaptic model parameters**.

**Name**	**Value**	**Source**
τ^MSN_*D*1_ − SNr^_gaba_	5.2 ms	Connelly et al., 2010
*g*^MSN_*D*1_ − SNr^_0_	2 nS	constrained by Connelly et al. (2010)
*t*^MSN_*D*1_ − SNr^_delay_	7 ms	Connelly et al., 2010
*E*^MSN_*D*1_ − SNr^_rev_	−80 mV	Connelly et al., 2010
τ^GPe − SNr^_gaba_	2.1 ms	Connelly et al., 2010
*g*^GPe − SNr^_0_	76 nS	Connelly et al., 2010
*t*^GPe − SNr^_delay_	3 ms	Nakanishi et al., 1991
*E*^GPe − SNr^_rev_	−72 mV	Connelly et al., 2010
τ^STN − SNr^_ampa_	12 ms	n.d. assume as for STN to GPe Hanson and Jaeger (2002)
*g*^STN − SNr^_0_	0.91 nS	fitted to model constrains and in range of Shen and Johnson (2006)
*t*^STN − SNr^_delay_	4.5 ms	Shen and Johnson (2006) and Ammari et al. (2010)
*E*^STN − SNr^_rev_	0 mV	n.d.
τ^MSN_D2_ − GPe^_gaba_	6 ms	Shen et al., 2008
*g*^MSN_*D*2_ − GPe^_0_	2 nS	constrained by Shen et al. (2008)
*t*^MSN_*D*2_ − GPe^_delay_	7 ms	Park et al., 1982
*E*^MSN_*D*2_ − GPe^_rev_	−65 mV	Rav-Acha et al., 2005
τ^STN − GPe^_ampa_	12 ms	Hanson and Jaeger, 2002
*g*^STN − GPe^_0_	0.35 nS	fitted to model constrains and in range of Hanson and Jaeger (2002)
*t*^STN − GPe^_delay_	5 ms	Ammari et al., 2010
*E*^STN − GPe^_rev_	0 mV	n.d.
τ^GPe − GPe^_gaba_	5 ms	Shen et al., 2008
*g*^GPe − GPe^_0_	1.3 nS	fitted to model constrains and in range of Hanson and Jaeger (2002)
*t*^GPe − GPe^_delay_	1 ms	n.d.
*E*^GPe − GPe^_rev_	−65 mV	n.d. assumed as for MSN D1
τ^CTX − STN^_ampa_	4 ms	Baufreton et al., 2005
*g*^CTX − STN^_0_	0.25 nS	n.d.
*t*^CTX − STN^_delay_	2.5 ms	Fujimoto and Kita, 1993
*E*^CTX − STN^_rev_	0 mV	n.d.
τ^GPe − STN^_gaba_	8 ms	Baufreton et al., 2005
*g*^GPe − STN^_0_	0.08 nS	n.d. fitted to model constrains
*t*^GPe − STN^_delay_	5 ms	Baufreton et al., 2005
*E*^GPe − STN^_rev_	−84 mV	Baufreton et al., 2009

To estimate the connectivity between GPe and SNr we use the following:

1. GPe axons form baskets around target SNr neurons giving rise to multiple large synaptic boutons (Smith et al., 1998) and activation of a single GPe neuron evokes large IPSPs with a conductance estimated as 76 nS (Connelly et al., 2010). This indicates that the GPe neurons exert a strong inhibitory control over SNr neurons through multiple synaptic contacts on the GPe neuron.
2. Pharmacologically induced inhibition of GPe leads to a large increase of firing rate at more than 300% of basal SNr activity (Celada et al., 1999). We tuned the SNr neuron in the network, by injecting current (254 pA) and adding STN input (at 10 Hz), to fire at above 300% of GPe base firing rate without input from GPe. Note that STN activity have been reported to increase to 20 Hz without GPe input (Farries et al., 2010), thus maintaining STN at 10 Hz might seem to be the wrong thing to do. However, experiments (Moran et al., 2011; Rosenbaum et al., 2012a) and model predictions (see Results below) suggest that the synapses between STN and SNr are depressing. Thus, when tuning the model with static synapses between STN and SNr we did not change the activity of STN in order to avoid overestimating the effect of STN to SNr. We found that emulated input from 32 GPe neurons, each with firing frequency around 30 Hz and depressive synapses with 76 nS (Connelly et al., 2010) as the max conductance strength, were needed to decrease the firing rate of the SNr neuron close to 30 Hz.

To estimate the connectivity between GPe and STN we use the following:

1. GPe has sparse but selective connectivity with STN, with an estimate of 300 synapses per GPe neuron (Baufreton et al., 2009). It has also been estimated that single GPe axons make multiple synaptic contacts with one STN cell (Smith et al., 1990).
2. We assume that a single GPe makes on average 10 synaptic contacts with each STN, then, given 1 above, we estimate that each GPe makes 30 connections in STN.
3. STN neurons increase their firing rate with 100% when removing GPe input (Farries et al., 2010) whereas GPe firing rate decrease with 50% when removing STN input (Féger and Robledo, 1991). The synaptic weight between GPe and STN was tuned such that this was fulfilled.

To estimate the connectivity between STN to GPe and SNr we use the following:

1. STN terminals spread evenly over perikarya and dendrites of GPe and SNr neurons (Smith et al., 1998), and have a synaptic conductance around 1 nS for GPe and SNr (Hanson and Jaeger, 2002; Shen and Johnson, 2006).
2. STN fires at 10 Hz *in vivo* (Fujimoto and Kita, 1993; Paz et al., 2005; Walters et al., 2007; Farries et al., 2010), and silencing the nucleus leads to a 50% decrease of activity in GPe and SNr (Féger and Robledo, 1991).
3. Assuming that STN neurons make 30 connections in GPe or SNr we found that with the synaptic weights at 0.35 nS and 0.91 nS for respectively STN to GPe and STN to SNr connections we were in range of criteria 1 and fulfilled criteria 2.

The MSN D2 type makes synaptic contact preferentially on distal dendrites in GPe similarly to MSN D1 in SNr (Smith et al., 1998). Given that MSNs innervate their target in a similar way we assumed that the number of connections between MSN D2 and GPe equal the number of connection between MSN D1 and SNr. Estimation of GPe collaterals:

1. GPe collaterals innervate the soma and proximal dendrites, transmitting information reliably (Sadek et al., 2007; Sims et al., 2008) with an estimate around 500 synapses per neuron (Sadek et al., 2007).
2. We assume that a single GPe neuron makes on average 17 synaptic contacts with each GPe, then, given 1 above, we estimate that each GPe neuron makes 30 connections in GPe.
3. GPe firing rate increases with 55% when the MSN and collateral GPe inputs are removed (Celada et al., 1999).
4. We found that 3 was fulfilled with the conductance of pallidal synapse set to 1.3 nS, which is in line with Sims et al. (2008).

Estimation of synaptic input rate between cortex and STN:

1. STN neurons fire at around 10 Hz *in vitro* (Fujimoto and Kita, 1993; Paz et al., 2005; Walters et al., 2007; Farries et al., 2010).
2. Without inhibitory input STN neurons fire at 20 Hz (Farries et al., 2010).
3. Assuming a conductance of 0.25 nS we set the cortical input rate to 189 Hz to fulfill 1 and 2.

The resulting connectivity parameters are listed in Table 4 and the mentioned synaptic conductances in Table 5. See Figure 1G for the effect on network base firing rate following different lesions.

### Synapse models

In order to reveal how activity dependent synapses differentially shape post-synaptic neuron firing frequencies, all simulation results are also compared with the case when static reference (i.e., frequency independent) synapses are used instead. To model the simpler static synapse, a standard conductance based exponential decay model (Equation 4) is used.

(4) $$\frac{dg}{dt}=-\frac{g}{\tau_{\text{syn}}}+g_{o}\times \delta (t-t_{\text{spike}})$$

Here *g* is the conductance, τ_syn_ (syn = ampa/gaba) the synaptic time constant, *g*_*o*_ the maximal conductance for a synaptic event, *t*_spike_ the time of the synaptic event and δ is the Dirac delta function. When a pre-synaptic spike arrives, the conductance *g* is updated with *g*_0_ and then, in between the spikes, the conductance decays toward zero with time constant τ_syn_. The post-synaptic current is given by *I*_syn_ = *g* × (*E*_rev_ − *V*).

To model a frequency dependent synapse, the Tsodyks model (Tsodyks et al., 1998) was used (Equations 5 and 6) with the common FD formalism (Abbott et al., 1997; Dittman et al., 2000; Abbott and Regehr, 2004; Puccini et al., 2007). The FD formalism dictates that the synaptic strength is updated by the product of facilitating (F) and depressing (D) variables/factors. This description shows quantitatively good approximations of experimentally measured synapse dynamics (Tsodyks and Markram, 1997; Markram et al., 1998; Planert et al., 2010; Klaus et al., 2011). The model formalism assumes a finite pool of synaptic resources in active (*y*), inactive (*z*) and recovered (*x*) states. At rest *y* and *z* are 0 and *x* is 1. Depression occurs because some of the resources remain for a while in the inactive state before entering the recovered state with a rate determined by the recovery time constant τ_rec_. The facilitation is modeled by *u* which is a variable that is step-wise increased at each spike with the product of the utilization factor *U* and 1 − *u* (*U* is between 0 and 1) and decays exponentially toward 0 with time constant τ_fac_ in between spikes (Equation 5). The resources in the active state *y* are increased with the product of the variables *x* and *u* (capturing depression and facilitation respectively) and are then quickly inactivated by decaying toward zero with time constant τ_syn_ (Equation 6). The post-synaptic conductance is proportional to the fraction of resources in the active state and is given by *g* = *g*_0_ × *y* with the resulting post-synaptic current *I*_syn_ = *g* × (*E*_rev_ − *V*).

(5) $$\frac{du}{dt}=-\frac{u}{\tau_{\text{fac}}}+U\times (1-u)\times \delta \left(t-t_{\text{spike}}\right)$$

(6) $$\begin{array}{l} \frac{dx}{dt}=\frac{z}{\tau_{\text{rec}}}-u\times x\times \delta (t-t_{\text{spike}})\\ \frac{dy}{dt}=-\frac{y}{\tau_{\text{syn}}}+u\times x\times \delta (t-t_{\text{spike}})\\ \frac{dz}{dt}=\frac{y}{\tau_{\text{syn}}}-\frac{z}{\tau_{\text{rec}}} \end{array}$$

The value and source of the basic synaptic parameters, τ_syn_ (syn = ampa/gaba), *g*_*o*_, *t*_delay_ and *E*_rev_, for both plastic and static synapse models are listed in Table 5. In simulations the synaptic weights and delays were randomly drawn from a uniform interval ±50% of peak conductances *g*_0_ and delays *t*_delay_. We created two static reference synapses from MSN D1 data; a weak static synapse ref^MSN_*D*1_^_init_ representing the initial non-facilitated peak conductance, *g*^MSN_*D*1_ − SNr^_0_, and a strong static synapse ref^MSN_*D*1_^_max_ representing the maximally facilitated peak conductance, 4 × *g*^MSN_D1_ − SNr^_0_, during steady-state (see also Figure 1I). The unitary conductive strength *g*^MSN_D1_ − SNr^_0_ of a striato-nigral synapse could not be established by Connelly et al. (2010). From their data we however, estimate the conductance to 2 nS, assuming it to be 50% of the measured mean conductance strength evoked by minimal stimulation of MSNs inputs. The mean conductance was calculated by dividing the measured peak of the first inhibitory post-synaptic current, 300 pA, with the driving force, 75 mV (GABA high chloride reversal potential at 5 mV and holding potential is at −70 mV). For GPe we have one reference synapse ref^GPe^_30 Hz_ with conductance 0.15 × *g*^GPe − SNr^_0_ which is the steady-state strength of the depressing synapse at 30 Hz activation (a typical *in vivo* frequency). The unitary conductive strength of *g*^GPe − SNr^_0_ was set to 76 nS as measured by Connelly et al. (2010). The static synapse STN synapse in SNr was named *ref*^*STN*^ and had the synaptic strength *g*^STN − SNr^_0_. In Figure 1H are the dynamics of the static synapses onto SNr displayed.

For facilitating and depressing synapses in SNr we use two data sets collected from the published material by Connelly et al. (2010) for tuning of the synapse models. The first data set describes the relative synaptic current increase over 10 successive spikes at 10, 50, and 100 Hz and the second data set shows the relative size of a recovery spike after 5 pulses at 100 Hz and measured after 60, 160, 560, 3000, and 9000 ms. For facilitating synapse in GPe we used one data set from Sims et al. (2008) with the relative synaptic current increase over 10 successive spikes at 20 and 50 Hz. We fitted parameters for the Tsodyks synapse in Matlab using a least square method minimizing the squared error between experimental and model current pair pulse data. To find the solution we used the *fminserach* method in Matlab which implements the Nelder-Mead Simplex method (Lagarias et al., 1998). The resulting parameters for the facilitating MSN D1 synapse, fac^MSN_*D*1_^, and depressing GPe synapse, dep^GPe^, in SNr, and facilitating MSN D2 synapse, fac^MSN_D2_^, in GPe, are listed in Table 6 and the resulting behavior of the dynamic synapses onto SNr, fac^MSN_*D*1_^and dep^GPe^, are displayed in Figures 1I–K. The weights of the dynamical synapses were tuned such that the conductance of the first spike equaled *g*^MSN_*D*1_ − SNr^_0_ and *g*^MSN_*D*2_ − GPe^_0_ for the MSN synapses onto SNr or GPe, and *g*^GPe − SNr^_*o*_ for the GPe depressing synapse onto SNr. Finally Moran et al. (2011) and Rosenbaum et al. (2012b) suggest that STN connects with depressing synapses to the basal ganglia output nucleus SNr. For the STN synapse in SNr we assumed standard depressing synaptic parameters (Tsodyks and Markram, 1996) with *U* = 0.35 and τ_ref_ = 800, with a peak conductance of 3.64 × *g*^STN − SNr^_*o*_. This ensured that the synaptic efficacy of the depressing STN synapse, at 10 Hz activation, was equal to *g*^STN − SNr^_*o*_.

**Table 6:** **Parameters for facilitating and depressing Tsodyks synapse models**.

**Synapse**	***U***	**τ_rec_ (ms)**	**τ_fac_ (ms)**
fac^MSN_*D*1_^	0.0192	623	559
dep^GPe^	0.196	969	0
fac^MSN_*D*2_^	0.24	11	73
dep^STN^	0.35	800	0

### Definition of “threshold coding” and “rate coding” in SNr used in this study

Striatal MSNs show firing rate changes with respect to the behavioral choice or according to the reward or the reward expectancy for certain actions (Ito and Doya, 2009). SNr neurons likewise change their activity and are modulated by duration and contingency of actions (Fan et al., 2012). Neurons in SNr can potentially code for action on/off or for a graded action-value/salience. In tasks where the basal ganglia are assumed to be involved in action selection (Albin et al., 1989; DeLong, 1990; Mink, 1996; Redgrave et al., 1999) an action is selected when a threshold is passed and consequently an action is either on or off. We call this *“threshold coding”* and in accordance with earlier work, we define that an action is signaled/selected as the firing rate of an SNr neuron drops below 5 Hz (Chevalier and Deniau, 1990; Humphries et al., 2006). Furthermore the basal ganglia might play a role in coding for different action-values (Samejima et al., 2005) or action saliences (Redgrave et al., 1999). Studies in monkeys suggest that action-value, independent of resulting actions, is coded in the firing rate of striatal neurons (Samejima et al., 2005; Lau and Glimcher, 2007, 2008; Pasquereau et al., 2007). Also SNr neurons show graded increases and decreases in firing rate in relation to action duration and likelihood (Fan et al., 2012). We call this *“rate coding*” and we thus also investigate how well changes in input rates, filtered by activity dependent synapses, can be picked up in the output nuclei.

### Implementation

The simulations were run using the NEST simulator (Gewaltig and Diesmann, 2007) and the network was built using PyNest which is a Python-interface to the NEST simulator. Model fitting of dynamical synapses were done in Matlab. The scripts necessary to run the model are available for download at ModelDB (http://senselab.med.yale.edu/ModelDB/).

## Results

### Characteristics of the derived model neurons and their synaptic inputs

The SNr, GPe and STN neuron models were tuned to exhibit properties that are characteristic of the firing of these neurons *in vitro*, exhibiting realistic membrane resistances (Figure 1A) and current frequency relationships (Figure 1B). The SNr neuron model was tuned to exhibit a switch from silence to spiking above 1 Hz at −54 mV (Figure 1C upper panel) and in addition it showed hyperpolarization induced rebound spikes (Figure 1C lower panel). The GPe neuron exhibited noise induced oscillations close to spike threshold (Figure 1D first trace), and then fired regular at higher current input intensities (Figure 1D second trace). It also showed rebound spikes upon release from hyperpolarization (Figure 1D third trace). The STN neuron model mimics the characteristic hyperpolarization induced burst, where the length of the burst depends both on the duration (Figure 1E first-third trace) and the magnitude (Figure 1E fourth-sixth trace). It also showed a dependency on time to first spike after a depolarizing 500 ms current induced high frequency discharge (Figure 1E seventh trace). To get the spontaneous activity seen in *in vitro* experiments for the SNr (7–20 Hz), the GPe (7–17 Hz), and the STN (8–12 Hz) neuron model, the parameter *I*_*in vitro*_ (see Tables 1–3) was respectively set to 15, 5, and 6 pA.

Synaptic conductances in the model (Table 5) where picked such that they would be in agreement with *in vitro* experiments. A few of the parameters in the model were tuned (see Materials and Methods) within biological realistic ranges, such that the steady-state firing rate of SNR, GPe and STN populations in control and lesion experiments were in agreement with literature (Figures 1F,G). The model of the facilitating striato-nigral and striato-pallidal, and depressing pallido-nigral synapses are fitted to data from *in vitro* experiments (Table 6). The dynamics of the plastic synapse types onto SNr is shown in Figures 1I–K. The facilitating MSN D1 to SNr synapse with peaking synaptic steady state strength at 10 Hz is around four times the resting state (base) conductance (Figure 1I), and a fast depressing GPe-SNr synapse which at 30 Hz has a steady state conductance around 15% of the resting state base line (Figure 1J). Depressing STN synapses in SNr were assumed to have standard depressing synaptic parameters (Tsodyks and Markram, 1996). Our full model constituted a network of SNr, GPe, and STN neurons, with connection parameters listed in Table 4, and the network was activated with emulated patterns of activity from respectively MSN D1, MSN D2, and Cortex (Figure 1L).

### Delayed SNr inhibition due to synaptic facilitation in the direct pathway

**Figure 2:**
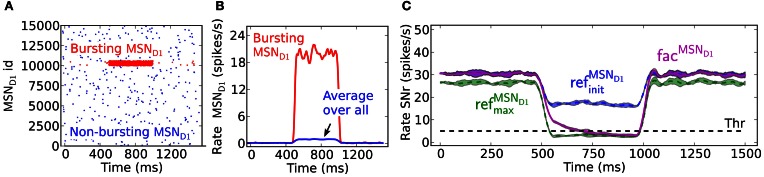
**The delayed effect of facilitating synapses on MSN D1 to SNr inhibition. (A)** Raster plot of the emulated activity of 15,000 pre-synaptic MSNs with 4% of the neurons bursting (red) at 20 Hz for 500 ms and the rest of the population (blue) firing at 0.1 Hz. **(B)** Firing frequency of pre-synaptic MSNs shown in **(A)** averaged over the whole population (blue), and over the bursting inputs (red) (triangular kernel window 100 ms used). **(C)** The resulting inhibitory response in SNr over time. The fac^MSN_*D*1_^ synapses (magenta) need time to be fully activated, delaying the threshold crossing for 200 ms here. With the ref^MSN_*D*1_^_init_ (blue) and ref^MSN_D1_^_max_ (green) synapses the inhibitory effect appears immediately (triangular kernel window 100 ms). The standard deviation of population activity between simulations is shown as shaded areas around the mean (solid or dotted lines).

The presence of facilitating synapses in the striato-nigral pathway can significantly delay the suppression of SNr firing following activation of only a few pre-synaptic MSNs spiking at moderate burst frequency. The decrease in the SNr firing rate and the temporal changes during the burst period differ when the input arrives through the static ref^MSN_*D*1_^_init_, ref^MSN_D1_^_max_ vs. fac^MSN_*D*1_^ synapses (Figures 2A–C). In the example, 4% of the MSNs are bursting at 20 Hz. If assuming threshold coding, the threshold passing occurs in the simulations with the ref^MSN_D1_^_max_ and facilitating synapse model, whereas with the ref^MSN_*D*1_^_init_ synapse model the SNr neuron is not effectively suppressed. The facilitating synapse in the striato-nigral pathway needs, however, about 200 ms before it reaches the same conductive strength as when the ref^MSN_D1_^_max_ static synapse is used. Threshold passing is thus delayed for 200 ms when only a few pre-synaptic MSNs are active, showing that the communicated inhibitory signal is successively increasing over time before it suppresses the SNr neuron.

### Synaptic depression in the indirect pathway allows detection of irregular GPe activity

**Figure 3:**
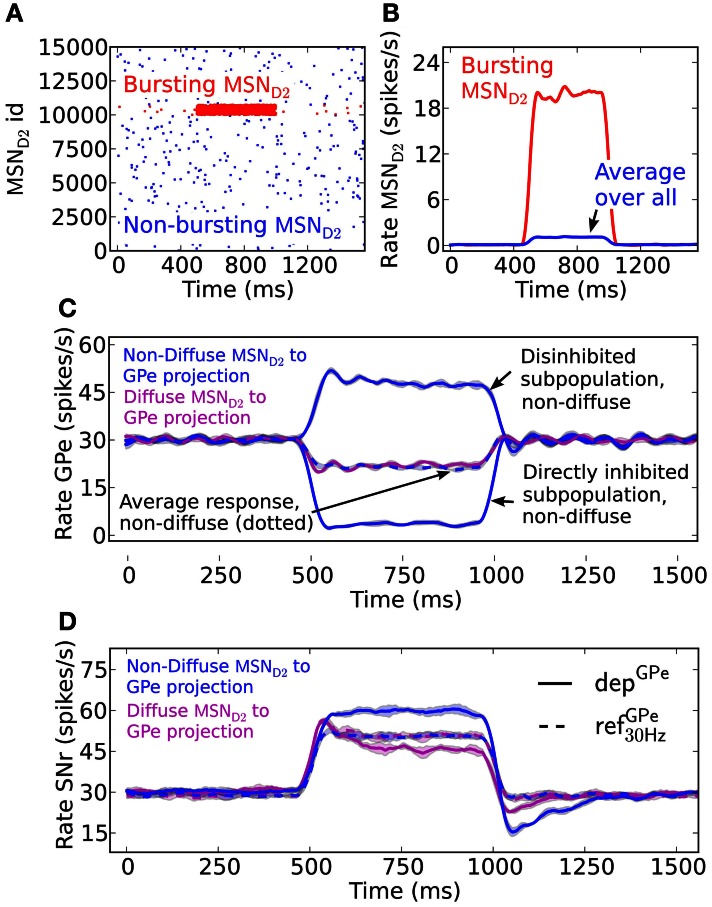
**The effect in SNr of depressing GPe to SNr synapses following activation of the indirect pathway. (A)** Raster plot of a population of 15,000 MSN D2 with 5% neurons bursting (red) at 20 Hz for 500 ms and the remaining population (blue) firing at 0.1 Hz. **(B)** Firing frequency of MSN D2 input populations bursting- (red) and total population (blue) (triangular kernel window 100 ms). **(C)** Firing frequency of the GPe population when they are assumed to be diffusely inhibited by the whole pre-synaptic MSN D2 pool (magenta) and firing frequency of the GPe population when a non-diffuse (topographic) MSN D2 to GPe projection is assumed (blue). This results in some (almost) pausing GPe neurons and some with increased firing. Note that together the GPe neurons have the same average firing rate change as the diffusely inhibited population (blue dotted) (triangular kernel window 100 ms used). The standard deviation of population activity between simulations is shown as shaded areas around the mean (solid or dotted lines). **(D)** Resulting disinhibition in SNr when the pre-synaptic GPe neurons receive non-diffuse or diffuse inhibition from MSN D2, magenta vs. blue in **(C)** for depressing (solid lines) and static (dotted lines) synapses. When the pre-synaptic GPe neurons are diffusely inhibited (magenta) the spike elevation in SNr is decreasing over time with depressing GPe to SNr synapses (magenta solid line) in contrast to when static synapses are used (magenta and blue dotted lines). The disinhibition of SNr via the indirect pathway is most efficient when the GPe projections are assumed to be non-diffusely inhibited such that the GPe has pausing subpopulations (blue solid line) (triangular kernel window 100 ms). The standard deviation of population activity between simulations is shown as shaded areas around the mean (solid or dotted lines).

A burst in MSN D2 subpopulations is most effective in disinhibiting SNr when this leads to pauses in GPe subpopulations (Figures 3A–C). GPe neurons have a peculiar firing pattern *in vivo*. They fire tonically at high frequency around 30 Hz *in vivo*, interrupted by bursts and pauses (Jaeger and Kita, 2011; Kita and Kita, 2011). During dopamine depleted condition the number of bursts and pauses increase, but still the same mean firing rate is maintained. The increased irregular activity of GPe neurons under dopamine depleted conditions have been hypothesized to disturb the information processing in basal ganglia output nuclei (Kita and Kita, 2011). Here we investigate how depressing GPe synapses convey the irregular GPe activity to SNr. We test this by setting up two scenarios. The first scenario is when both the pre-synaptic bursting and non-bursting MSN D2 subpopulations project in a diffuse way to all post-synaptic GPe neurons, such that the population of GPe neurons only sense the average change of MSN input (Figure 3C). A burst in an MSN D2 subpopulation then leads to a minor homogenous decrease in the GPe population. Simulations show that the resulting disinhibition in SNr will be stronger with static synapses, ref^GPe^_30 Hz_, than with depressing, dep^GPe^, synapses (Figure 3D) because the depressing GPe synapses in SNr recover their inhibitory strength over time as a result of the decreased GPe spike frequency, and thus the firing rate in SNr is higher in the beginning of the burst. Thus, in this scenario depressing synapses are responsible for producing a transient disinhibition of SNr following a burst in MSN D2. The second scenario is when striatal bursting and non-bursting MSN D2 project in a non-diffuse way (i.e., topographic) to post-synaptic GPe neurons. Here the GPe neurons receiving input from the bursting pre-synaptic MSN D2 become almost silent and the GPe population receiving input from the non-bursting pre-synaptic MSNs increase their firing further (due to reduced inhibition from the directly inhibited GPe neurons) (see Figure 3C). This situation is more effective in disinhibiting SNr over the whole burst (Figure 3D), even though the number of synaptic events/s from the total pool of pre-synaptic GPe neurons are the same as above (Figure 3C solid magenta vs. dotted blue line). The explanation is that the synapses of the subpopulation of the already tonically firing GPe neurons, which further increase their firing, become even more depressed and therefore do not compensate for the removed inhibition from the subpopulation which becomes quiet. Note that when the MSN D2 to GPe inhibition suddenly is released the synapses of the previously silenced GPe subpopulation have recovered in strength and are responsible for a transient inhibitory response in SNr (see discussion for a hypothetical effect of this). The present simulations thus indicate that irregular activity in GPe subpopulations leads to increased spiking in SNr despite no change in GPe to SNr mean synaptic activation frequency. This might contribute to the disturbed signaling through the basal ganglia output nuclei during Parkinson's disease.

### Detection of MSN D1 bursting subpopulations in the direct pathway

**Figure 4:**
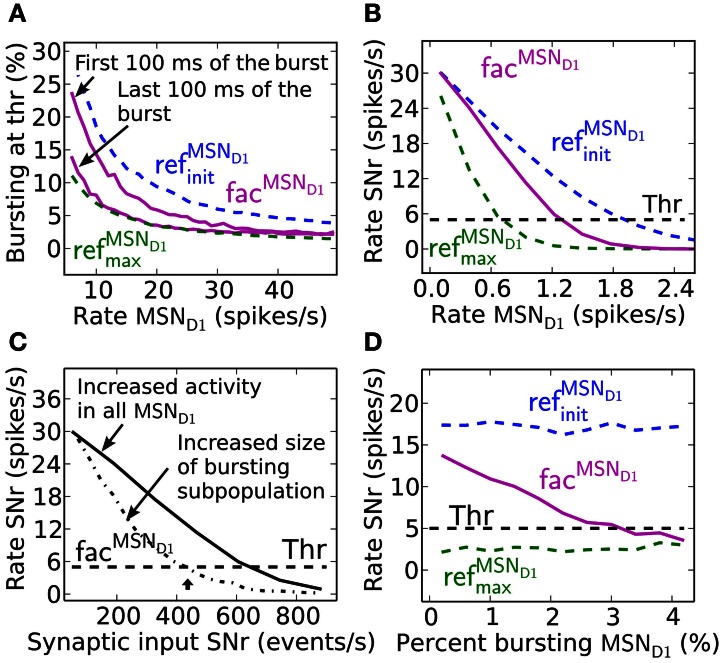
**Effects of synaptic facilitation in the direct pathway during steady-state. (A)** The number of MSN D1 bursting with a certain frequency (7–48 Hz) which are needed for action selection, defined as decreasing SNr firing under a certain threshold. If facilitated synapses are used (magenta), only a few MSNs are needed when bursting in the interval 17–48 Hz, and with performance closer to ref^MSN_D1_^_max_ (green) synapses than to ref^MSN_*D*1_^_init_ (blue) synapses during the last 100 ms of the 500 ms burst. **(B)** Steady-state firing rate in post-synaptic SNr cells when all pre-synaptic MSN D1 successively increase their firing. Facilitating synapses (magenta) allow background activity to increase up to 1.2 Hz before suppressing SNr to action signal threshold. **(C)** SNr neuron activity when increasing the total number of MSN D1-SNr synaptic events (#/s). Significantly fewer synaptic events are necessary to bring SNr below threshold if the pre-synaptic inputs come from a subpopulation of bursting MSN D1. Arrow corresponds to the synaptic event intensity used in Figure 2C. **(D)** Example of SNr activity as a function of number of bursting pre-synaptic MSN D1 when keeping the total number of synaptic events constant (450 events/s). The facilitating synapse (magenta) enables the SNr neuron to detect a change in input patterns resulting from a few bursting MSN D1.

Facilitating synapses selectively enhance input arriving at high frequency rates as in *in vivo* experiments. This is likely important because the number of simultaneously bursting MSNs in striatum is estimated to be low at any given time point (Wilson, 1993). The activation of only a few percent of pre-synaptic direct pathway MSNs, which burst with physiologically realistic burst frequencies, 17–48 Hz (Miller et al., 2008), results in robust inhibition of SNr during steady-state (Figure 4A). At lower MSN D1 spike frequencies, action signaling, if assumed to require threshold coding, becomes more resource demanding requiring activation of significantly higher numbers of pre-synaptic MSNs. As indicated in Figure 2 above, facilitation increases the response to pre-synaptic signals over time, with the result that fewer neurons are required to sustain the same amount of inhibition if the burst is sustained a few 100 ms (Figure 4A). Synaptic facilitation thus enables signal amplification of sustained bursts in the striato-nigral pathway. Such amplification due to synaptic facilitation has also been observed in hippocampus (Klyachko and Stevens, 2006), where facilitating synapses enhance the input during epochs of high frequency discharge associated with hippocampal place fields, suggesting that this might be a general function of facilitating synapses.

Facilitating synapses filter out low frequency input possibly preventing unspecific modulation of SNr firing rate due to a fluctuation in background MSN D1 activity. Facilitating synapses stay weak (as for simulation with ref^MSN_*D*1__init_^) when activated at low input rates, limiting the inhibitory effect of such a signal (Figure 4B). Simulations suggest that threshold passing in SNr is not occurring with an increase in background activity of the whole pre-synaptic MSN D1 pool up to 1.2 Hz. Facilitating synapses thus disregard low frequency input and buffer effectively against fluctuations in the basal activity.

Another way to quantify how the facilitating synapses can detect high frequency input, but buffer against changes in background firing is illustrated in Figure 4C, where significantly fewer synaptic events/s (400 compared to 600 synaptic events/s) are required to suppress the SNr when the input is arriving though pre-synaptic subpopulations with high frequency discharge rather than an unspecific increase in MSN D1 firing rate in the whole striatal pool (arrow indicates the intensity used in Figure 2C).

The above results show that facilitating synapses enable the post-synaptic neuron to differentiate between bursting- and non-bursting MSN D1 activity patterns, even though there are a constant number of pre-synaptic events. Increasing the number of high frequency firing direct pathway MSNs, and at the same time decreasing the background firing rate of the rest of the MSN D1 pool, such that the number of synaptic events is kept constant in post-synaptic SNr neurons will give a constant total inhibitory effect if ref^MSN_*D*1_^_init_or ref^MSN_D1_^_max_ static synapses are assumed (blue and green Figure 4D). However, with facilitating synapses detection of the changed pre-synaptic firing pattern is seen as a decrease in SNr firing rate with increasing contrast in spike frequency between the pre-synaptic neurons (magenta Figure 4D).

### Effects of depressing STN-SNr and GPe-SNr synapses for signaling through the indirect and hyperdirect pathways

**Figure 5:**
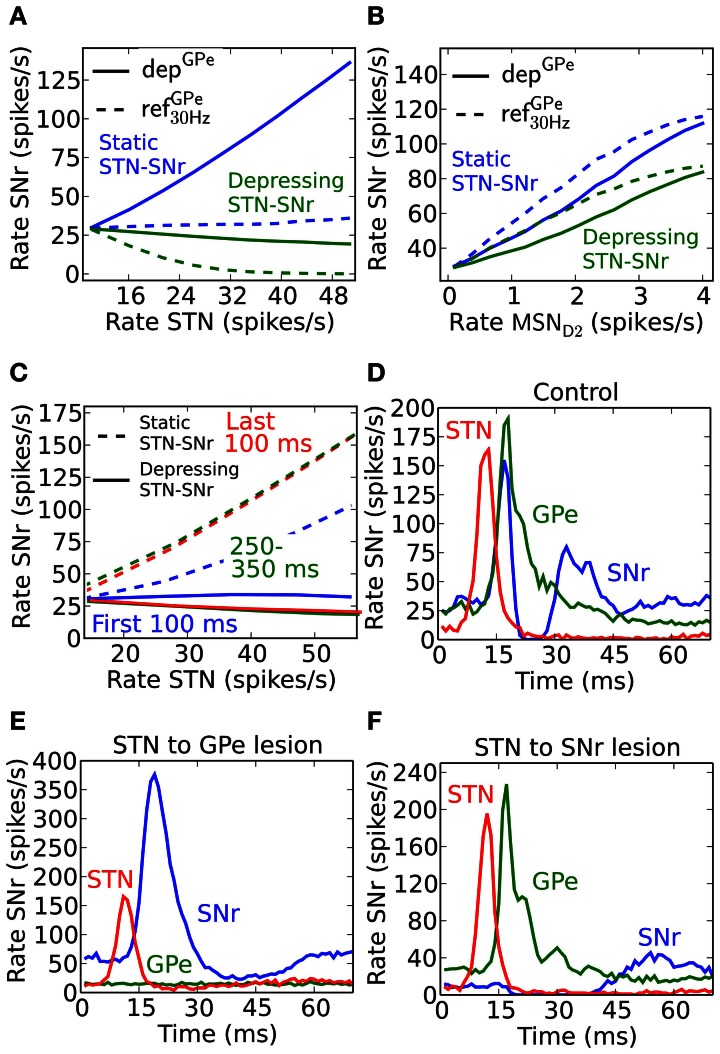
**Steady-state and temporal effects following activation of the indirect and hyperdirect pathway. (A)** Effects on SNr frequency when increasing the total STN population activity for dep^GPe^ (solid) and ref^GPe^_30 Hz_ (dotted) GPe to SNr synapses, and with static (blue) and depressing (green) STN-SNr synapses. **(B)** Effects on SNr frequency when increasing MSN D2 population activity for dep^GPe^ (solid) and ref^GPe^_30 Hz_ (dotted) synapses. **(C)** SNr activity in response to a 500 ms burst in STN during the first 100 ms (blue), between 250 and 350 ms (green) and during the last 100 ms (red) using depressing (solid) and static (dotted) STN synapses in SNr. **(D)** Rate in SNr (blue), GPe (green) and STN (red) after a brief (3 ms) high frequency excitatory pulse into STN. **(E)** Same as **(D)** but with STN to GPe lesioned. **(F)** Same as **(D)** but with STN to SNr lesioned.

An increased activity of STN may excite SNr directly and/or inhibit SNr through GPe. If both the GPe and STN synapses in SNr were static one would expect that they counteract each other, e.g., they might even cancel each other out such that increased activity in STN only leads to very small activity changes in SNr (Figure 5A, blue dotted line). But, since GPe synapses in SNr are depressing (Connelly et al., 2010), the activity from STN would come to dominate the response in SNr such that increased activity in STN leads to increased activity in SNr (Figure 5A, blue solid). This happens since depressing synapses tend to converge toward a constant post-synaptic current with increased firing rate (Tsodyks and Markram, 1996), thus the effect of the inhibitory signal through the depressing GPe-SNr synapses would saturate while the excitatory input from STN would continue to increase with frequency. Experimental studies in rat and monkey, however, contradict such scenarios, and rather suggest that increased activity in STN will not lead to increases in the basal ganglia output nuclei GPi, the analog to SNr (Maurice et al., 2003; Kita et al., 2005; Moran et al., 2011). Such results are well explained by published (Moran et al., 2011) and unpublished (Rosenbaum et al., 2012b) work suggesting that STN is assumed to connect to SNr with depressing synapses. With standard depressing STN-SNr synaptic parameters (Tsodyks and Markram, 1996) (Table 6) with *U* = 0.35, τ_fac_ = 0 and τ_rec_ = 800, our simulation results are in accordance with experimental results, i.e., that the excitatory control of SNr by STN is weak (Figure 5A, solid green). This suggests that STN is not a major contributor to increased activity in SNr if the input is channeled in parallel via GPe.

In contrast with the above prediction that steady state activation of the hyperdirect pathway leads to only small effects in SNr, the indirect pathway enhances SNr firing when activated from MSN D2 populations (Figure 5B). SNr is disinhibited in a (sub) linear fashion following sustained elevated MSN D2 background activity. Increased MSN D2 inhibition of GPe will indirectly increase STN firing through disinhibition, in turn increasing SNr firing significantly if STN-SNr synapses are static (Figure 5B, blue lines). When assuming depressing STN-SNr synapses a more moderate disinhibition through the indirect pathway is seen during steady state (Figure 5B, green curve).

From these results, achieved for steady-state activation of the hyperdirect and indirect pathways, one would predict that mainly the indirect pathway plays a significant role for controlling the SNr activity level. However, if the temporal effects are considered during e.g., different parts of a 500 ms burst, another scenario emerges. If assuming non-depressing STN-GPe synapses the STN input would indirectly excite SNr more and more during a 500 ms burst because of the GPe-SNr synaptic depression (solid lines in Figure 5C). We note, however, that with depressing synapses between both STN and SNr (Rosenbaum et al., 2012b) as well as between GPe and SNr the excitatory effect is not seen (dotted lines Figure 5C). The explanation is that the excitatory effect of the STN-SNr pathway is balanced by the inhibitory effect of the STN-GPe-SNr pathway.

To see an excitatory STN effect in the simulations when assuming both depressing STN-SNr and GPe-SNr synapses one needs to focus on an even finer time scale of a few tens of ms. The response following a very brief activation of STN generates a fast increase in activity followed by an inhibition and then a second increase in SNr (Figure 5D). This is in accordance with experiments in rat and monkey where such a triphasic response is evoked by a short pulse directly in STN or in cortex (Maurice et al., 2003; Kita et al., 2005; Jaeger and Kita, 2011). Note that in the simulations an activation of STN alone is sufficient to explain the triphasic response, even though the recruitment of the direct and indirect pathways are likely contributing during *in vivo* like conditions when stimulating in cortex. The inhibitory response in SNr following the brief STN activation can be extinguished by removing STN to GPe connections (Figure 5E), which also could be interpreted as if GPe and STN do not converge on the same post-synaptic SNr neurons and STN activation would excite those SNr populations over a longer time. The result is supported by experiments which show how application of Gabazine in GPi (homolog to SNr) in monkeys extinguishes the inhibitory and late excitatory response in GPi following cortical activation *in vivo* Tachibana et al. (2008). As expected, STN will indirectly inhibit SNr via GPe for a longer period when the connections between STN and SNr instead are removed (Figure 5F). This is also supported by experiments where blocking AMPA receptors in GPi in monkeys gives rise to a prolonged inhibition in GPi followed by a short period of elevated activity Tachibana et al. (2008). Simulations thus predict that for a brief activation of the hyperdirect pathway, a tri-phasic excitation-inhibition-excitation response pattern in SNr is seen if GPe and STN converge onto the SNr neurons. For longer STN bursts synaptic depression in both STN-SNr and GPe-SNr synapses prevents sustained effects in SNr. Thus, one could say that the presence of depressing synapses explain the somewhat puzzling experimental finding that STN for brief inputs excites SNr, but for longer activation has no effect or even decreases the firing rate in SNr (Maurice et al., 2003; Tachibana et al., 2008; Moran et al., 2011). Note that a burst in STN can still have a transient excitatory effect in SNr, controlled by the dynamics of the depressing STN synapses, if STN-SNr and STN-GPe-SNr pathways do not converge in SNr.

### Synaptic integration and neural coding in SNr

Striatal MSNs show firing rate changes with respect to the behavioral choice. Neurons which change firing rate according to reward probability for action candidates, are present in basal ganglia (Ito and Doya, 2009). SNr neurons likewise change their activity and are modulated by duration and contingency of actions (Fan et al., 2012). Neurons in SNr can thus potentially code for graded action-values/saliences (rate coding). To determine how synaptic facilitation and depression influence rate coding we quantify this by measuring the slope (Δ_SNr_/Δ_MSN_)of a linear fit to the frequency curves of MSN D1 or MSN D2 and SNr, and for different numbers of bursting MSNs. The slope factor indicates how well MSN input rates are sensed in SNr. A small slope factor shows that the activity level in SNr is only moderately controlled by the burst frequency of MSNs, whereas a large slope factor shows that MSN input frequencies are well represented in SNr.

**Figure 6:**
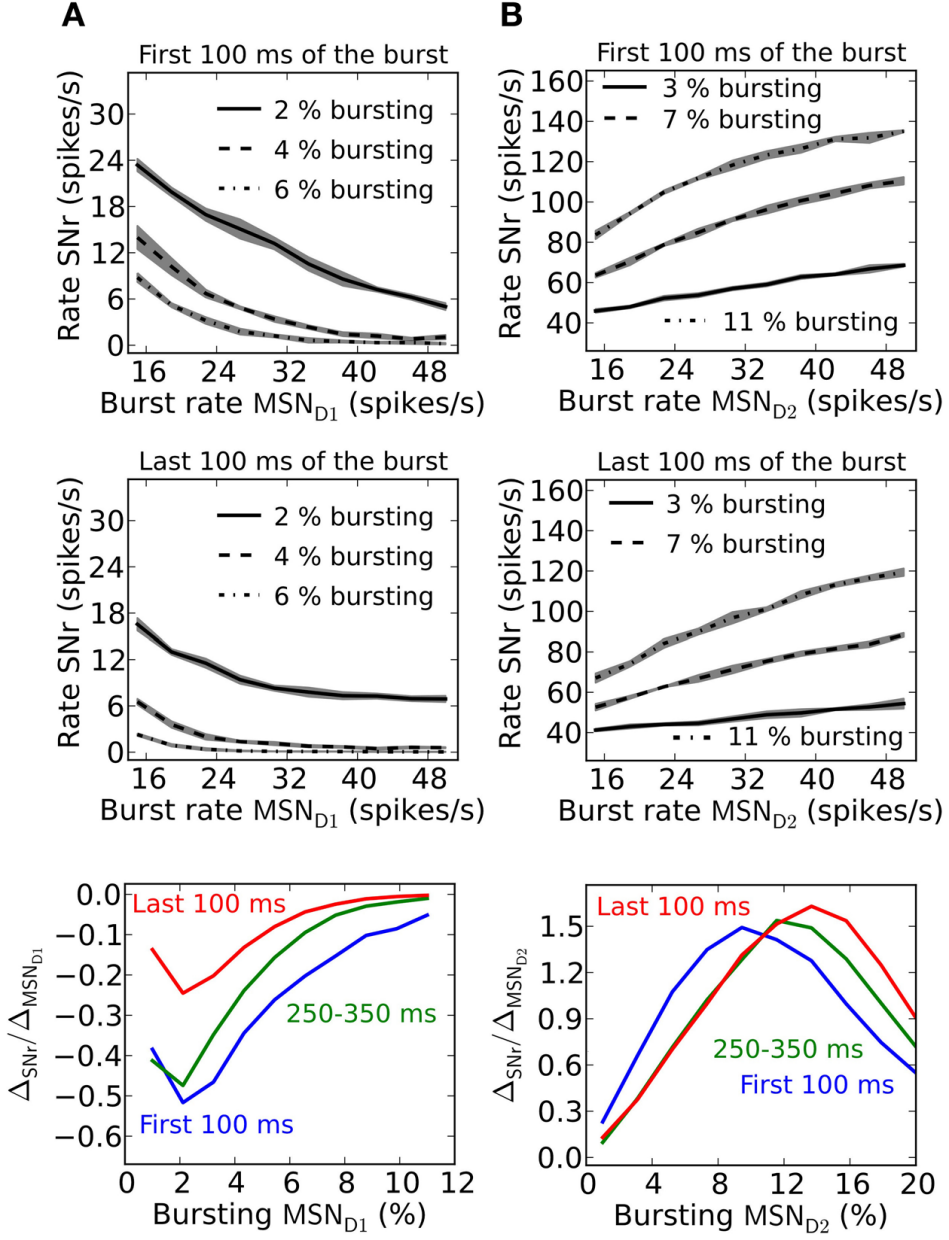
**Rate coding in SNr during a sustained burst in striatal populations. (A)** Upper panel; effect on SNr firing rate if 2, 4, or 6% of the pre-synaptic MSN D1 pool burst. The result is shown during the first 100 ms of a 500 ms long burst. Middle panel; same as upper panel but during the last 100 ms of the burst. Lower panel shows the slope of linear fits to traces such as in upper and middle panel for three intervals during a 500 ms burst: for the first 100 ms, between 250 and 350 ms and for the last 100 ms. The slope is plotted against the percent of bursting MSN D1. The standard deviation is shown as shaded areas around the mean. **(B)** Upper panel; effect on SNr firing rate if 3, 7, or 11% of the pre-synaptic MSN D2 pool is bursting. The result for the first 100 ms of a 500 ms long burst is shown. Middle panel; Same as upper panel but during the last 100 ms of the burst. Lower panel shows the slope of linear fits to traces such as in upper and middle panel for three intervals: the first 100 ms (blue trace), between 250 and 350 ms (green trace) and the last 100 ms (red trace). The result is plotted against percent of bursting MSN D2 populations. Diffuse MSN D2-GPe projections are assumed here (compare Figure 3). The standard deviation is shown as shaded areas around the mean.

Facilitating synapses allow better detection of MSN D1 firing rate changes in SNr during the first part of a burst (Figure 6A). This is further illustrated in the bottom panel in Figure 6A where the magnitude of the slope differ with a factor of 3 during the first 100 ms compared to the last 100 ms of a 500 ms burst. This result suggests that an MSN D1 subpopulation better signal rate coded action-values during an initial brief time window immediately following striatal activation. This is explained by the shape of the steady-steady activation curve of the facilitating MSN D1 synapse (Figure 1I). At longer time intervals the effective inhibition on SNr (spike frequency times the facilitation) levels off.

Depressing GPe-SNr synapses can enable rate coding of pre-synaptic MSN D2 populations during the whole burst interval (Figure 6B). The size of the pre-synaptic bursting MSN D2 population decides when such rate coding is most optimal. The optimal size of the MSN D2 subpopulation for rate coding is slightly increased over a 500 ms burst (Figure 6B, bottom panel).

### Co-activation of the direct- and indirect- or hyperdirect-pathway

**Figure 7:**
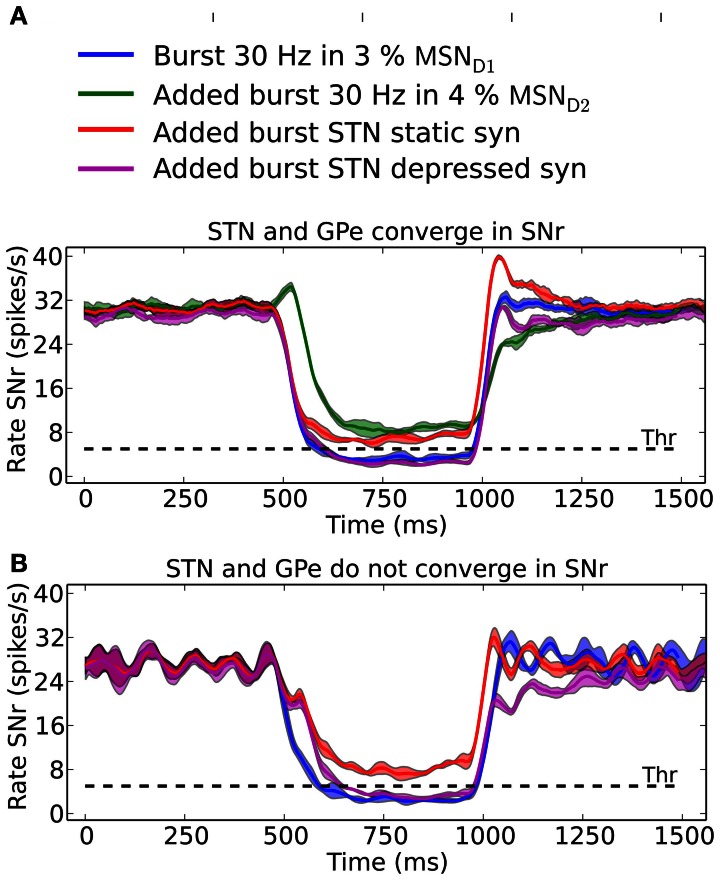
**The result of convergent and non-convergent striato-nigral, pallido-nigral and subthalmo-nigral inputs. (A)** Scenario when STN and GPe converge onto SNr neurons. 500 ms 30 Hz bursts in 3% of the MSN D1 pool: alone (blue), combined with 4% bursting MSN D2 (green), combined with elevated STN input induced by doubling the backround cortical drive to 2 × *v*_STN_ Hz and using static (red) or depressive STN to SNr synapses (magenta). The standard deviation is shown as shaded areas around the mean. **(B)** Scenario were STN and GPe do not converge in SNr. GPe recieves independent poisson input, instead of input from STN neurons, at 10 Hz. 500 ms 30 Hz bursts in 3% of the MSN D1 pool: alone (blue), combined with elevated STN input induced by doubling the backround cortical drive to 2 × *v*_*STN*_ Hz and using static (red) or depressive STN to SNr synapses (magenta). In **(B)** it is assumed that the SNr neurons measured from receive increased STN inputs in combination with a constant basal level of GPe inhibition. The standard deviation is shown as shaded areas around the mean.

SNr neurons increase and decrease their activity in relation to actions (Sato and Hikosaka, 2002; Basso and Sommer, 2011; Fan et al., 2012). SNr receives input from MSN D1, GPe, and STN and can potentially be decreased by either increased activity in striatal MSN D1 input or by increased activity in GPe input, whereas the activity in SNr can be increased either by disinhibition via GPe or by increased excitation from STN. It is not obvious which input is responsible for increases and decreases in activity in SNr seen in behavioral experiments (Fan et al., 2012). Our results suggest that it is the inhibitory input arriving from MSN D1 that is responsible for inhibition in SNr whereas di-synaptic input from STN through GPe only have a significant effect for very brief inputs (compare Figure 5). Conversely we found that MSN D2 can produce an increase in SNr activity through disinhibition via GPe, and that STN has only little effects on SNr activity. Combining inputs onto SNr we see how recruitment of MSN D2 can increase the activity in SNr, potentially suppressing an action signal initiated via MSN D1, especially during its initial phase of a 500 ms burst (Figure 7A, green line). Note that for a smaller proportion of bursting MSN D2 we would maybe get a delayed action signal when MSN D1-SNr synapses successively facilitate. A similar observation holds when the hyperdirect pathway is recruited. If the synapses between STN and SNr are assumed to be static (Figure 7A; red line) they counteract (or delay) an action selection signaling induced through the direct pathway. However, following our prediction that STN-SNr synapses are depressing then the excitatory control of SNr by STN is negligible (Figure 7A; magenta line, compare also Figure 5 above). Finally we tested how increased activity in STN influences SNr if GPe and STN do not converge in SNr neurons. Now, when simulating with depressing STN-SNr synapses onto the SNr neurons, we see (Figure 7B; magenta line) how STN can delay an action signal induced by MSN D1 activity for a period of 100–200 ms, a delay directly determined by the dynamics of the depressing synapses. Thus, the patterns of convergence of the direct, indirect and hyperdirect pathway determine the effect a signal though either of the pathways can have.

### How parameter pertubations influence the basal firing rates

**Figure 8:**
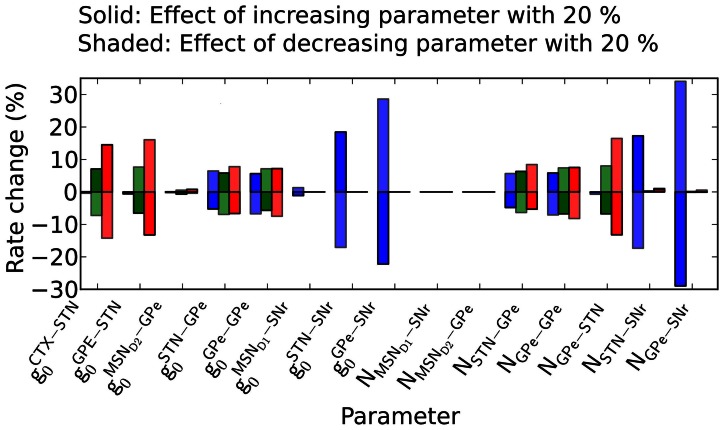
**Effect of increasing/decreasing parameter values on the steady-state activity in SNr, GPe, and STN**. Effects on firing rates in SNr, GPe, and STN when changing the number of incoming connections from each pre-synaptic neuron, *N*, or synaptic conductive strengths, *g*_*o*_, in the model. Here depressive STN-SNr synapses are assumed. Solid bars: the effect of a 20% increase in the parameter value indicated under the graph. Shaded bars: the effect of 20% decrease in the parameter values.

Simulations predict that parameter changes in GPe-SNr and STN-SNr connections affect the firing rate in SNr the most. The model have many parameters and one natural question is how robust the model behavior is to parameter changes. We tried to address this by varying the conductances and number of incoming connections from each pre-synaptic neuron with 20% up/down while measuring the change in the basal rates in SNr, GPe, and STN. We find that the rate in SNr is most sensitive to parameter changes in the pallido-nigral and subthalamo-nigral pathways (Figure 8) (*g*^GPe − SNr^_*o*_, *g*^STN − SNr^_0_, *N*_GPe − SNr_ and *N*_STN − SNr_). Specifically we see a superlinear change in firing rate in SNr when changing the paramters in the pallido-nigral pathway. The reason for the superlinear increase is the high inhibitory influence GPe has on SNr at basal firing rate. SNr neurons increases their firing rate with more than 300% (see Materials and Methods) when removing GPe (i.e., decreasing GPe activity with 100%), thus increasing the conductance or number of connections between GPe and SNr will have a strong effect. The firing rate in GPe and STN nuclei are significantly less effected and are more robust against changes in parameter values.

## Discussion

The present study has important implications for how to think about the role of basal ganglia pathways, and further contributes to the understanding of which combinations of pathways in basal ganglia are responsible for the signaling in basal ganglia output stages.

We have investigated how dynamical synapses in the direct, indirect and hyperdirect pathways quantitatively shape the activity in SNr neurons over time. The frequency dependencies of the synapses play a significant role in producing the response of SNr neurons to characteristic *in vivo* spike patterns from MSN D1, MSN D2, and cortex. Simulations predict that only bursting activity in a few percent of the direct or indirect pathways MSNs are sufficient to respectively substantially decrease or increase the activity in SNr. For the indirect pathways the model predict that, due to depressing synapses, irregular activity in GPe is more effective in increasing the SNr activity. We hypothesize that synapses between STN and SNr are depressing and thus could explain experiments showing that prolonged activation of STN has a weak effect on SNr firing rate whereas a brief STN input leads to a tri-phasic response in SNr. The prediction that STN-SNr synapses are depressing together with the result that GPe has a strong inhibitory control of SNr suggest that the signaling in the indirect pathway through either striatum-GPe-SNr or striatum-GPe-STN-SNr is functionally dominated by the former. Our findings further indicate that a rate code, signaling action-values or saliences, in striato-nigral pathways is optimal during the initial part of at 500 ms burst in a striatal subpopulations. For the indirect pathway the simulation showed that the input-output frequency separation could be obtained during most parts of the burst. Simulations suggest that for optimal rate coding only a low number of active pre-synaptic MSNs (a few percent) need to be activated in the direct and indirect pathways. We also show that facilitating MSN D1-SNr synapses enhance action signaling caused by increased activity in a small subpopulation of pre-synaptic MSN D1 and at the same time the presence of facilitating synapses buffer against non-specific action signaling due to fluctuation in striatal background activity. Likewise non-specific steady-state changes in background activity in MSN D2 are ignored as a result of depressing GPe-SNr synapses. In summary, the quantitative effects of the frequency dependent synapses on basal ganglia output stages seen in this study highlight the role of short term plasticity in the basal ganglia for signaling, and ultimately, for control of behavior.

In addition to controlling action selection, SNr also influences SNc. SNc provides the main dopaminergic input to the striatum and cerebral cortex. Loss of neurons in SNc is the major pathology behind the Parkinson's disease. Since a major source of GABAergic control of SNc is the neighboring SNr (Tepper and Lee, 2007), the temporal profile of activity in SNr, can effectively shape the activity of SNc over time. For example, our results suggest that when striatal inhibition is lifted from GPe, reactivated GPe synapses can inhibit SNr for a short interval since the GPe-SNr synapses are depressing. This transient inhibition of SNr may result in a short excitation in SNc. The duration of this activity (compare Figure 3D) in SNc (100–200 ms) is equal to the reported phasic dopaminergic signals (Redgrave and Gurney, 2006). Whether this chain of influence is at all involved in the generation of phasic dopamine signals is, however, to be elucidated in the future.

### Model assumptions

The qualitative results of the model are more robust to parameter changes compared to the quantitative results. For example, the finding that the detection of subpopulations of bursting or pausing neurons in the basal ganglia nuclei occurs while changes in background fluctuations are buffered against, are qualitative phenomena enabled by short-time plasticity. They are not dependent on the exact model connectivity or synaptic strength used. This also applies to the result of how short term plasticity in the pathways through the basal ganglia qualitatively shape the output signal over time. However, changes in parameters will e.g., affect the predicted proportion of striatal populations that need to be activated to significantly affect the basal ganglia output stages. Thus, to improve the quantitative properties of the model, it is necessary to successively update model parameters based on new data produced.

We have included important aspects of the basal ganglia circuitry with regard to the output stage, but in the present model the input from the striatum and cortex are emulated. By including GPe and STN we have tried to account for their important interactions. In future versions of the network it would be interesting to incorporate a striatal module and its interactions with GPe (Mallet et al., 2012).

Some recent papers have questioned the value of using a deterministic synapse model, and instead argued for moving to models which take into account the stochasticity of synaptic signaling (De la Rocha and Parga, 2005; Merkel and Lindner, 2010; Rosenbaum et al., 2012a). These studies showed that when one takes into account the trial-to-trial variability in synaptic release events, the resulting post-synaptic response can differ considerable on individual trials. However, considering that it is probably a population of neurons in basal ganglia output nuclei that are coding for a specific message, then averaging over the population likely represent the outcome. One future direction could, however, be to use a stochastic synaptic model and investigate how this affects the variability of signaling.

### The role of STN in basal ganglia

Several computational studies have tried to find the role for STN in basal ganglia signaling. Frank (2006) suggests that STN reduces premature behavioral responses by excitation of the basal ganglia output nuclei and thus dynamically adjusts the response threshold there. In Leblois et al. (2006) loops though STN-SNr/GPi-thalamus-cortex are assumed to compete with loops though striatum-SNr/GPi-thalamus-cortex in SNr/GPi, allowing the system to control action selection. In Humphries et al. (2006) inputs to STN have an excitatory effect in basal ganglia output nuclei setting an appropriate contrast level for action selection. All these models assume that activating STN results in increased activity in SNr. Experiments suggest that STN can control the firing rate in SNr following brief synchronized inputs, but not following prolonged activations. In reproducing these observations our simulations predict that STN makes depressing synapses in SNr. Our results further suggest that the effect STN can have on signaling in SNr depends on the convergence pattern of GPe and the exact dynamics of the synaptic depressions in GPe-SNr and STN-SNr synapses. We speculate that the hyperdirect pathway filter incoming signals such that transient brief signals are let through while longer sustained signals are disregarded. Brief excitations of SNr by STN could then possibly signal start or stop of actions. However, the role of such an STN filtering mechanism has to be settled by future work.

Recent work by Mallet et al. (2012) provides an alternative hypothesis for the role of STN in the basal ganglia network. Their study suggests that a subset of neurons in GPe are driven by STN, and each one of these GPe neurons in turn gives off over 10,000 GABAergic synapses in striatum and thus potentially have a significant inhibitory control of striatum. Thus, STN could serve an important role in regulating the activity of striatal neurons and gate the cortical and thalamic input activity at the striatal level. In line with the present study, such mechanisms of increasing or decreasing the number of activated striatal MSNs might significantly control signaling in basal ganglia output stages.

## Author contributions

Mikael Lindahl: Conception and design of research, performed simulations, analyzed data, interpreted results of simulations, prepared figures, drafted manuscript, edited and revised manuscript, approved final version of manuscript. Iman Kamali Sarvestani: Conception and design of research, interpreted results of simulations, drafted manuscript, edited and revised manuscript, approved final version of manuscript. Örjan Ekeberg: Conception and design of research, interpreted results of simulations, drafted manuscript, edited and revised manuscript, approved final version of manuscript. Jeanette Hellgren Kotaleski: Conception and design of research, interpreted results of simulations, analyzed data, drafted manuscript, edited and revised manuscript, approved final version of manuscript.

### Conflict of interest statement

The authors declare that the research was conducted in the absence of any commercial or financial relationships that could be construed as a potential conflict of interest.
